# Development of the First Cisgenic Apple with Increased Resistance to Fire Blight

**DOI:** 10.1371/journal.pone.0143980

**Published:** 2015-12-01

**Authors:** Thomas D. Kost, Cesare Gessler, Melanie Jänsch, Henryk Flachowsky, Andrea Patocchi, Giovanni A. L. Broggini

**Affiliations:** 1 Plant Pathology, Institute of Integrative Biology (IBZ), ETH Zurich, Zurich, Switzerland; 2 Agroscope, Institute for Plant Production Sciences, Wädenswil, Switzerland; 3 Julius Kühn-Institut (JKI), Federal Research Centre for Cultivated Plants, Institute for Breeding Research on Fruit Crops, Dresden, Germany; Virginia Tech, UNITED STATES

## Abstract

The generation and selection of novel fire blight resistant apple genotypes would greatly improve the management of this devastating disease, caused by *Erwinia amylovora*. Such resistant genotypes are currently developed by conventional breeding, but novel breeding technologies including cisgenesis could be an alternative approach. A cisgenic apple line C44.4.146 was regenerated using the cisgene *FB_MR5* from wild apple *Malus* ×*robusta* 5 (*Mr*5), and the previously established method involving *A*. *tumefaciens*-mediated transformation of the fire blight susceptible cultivar ‘Gala Galaxy’ using the binary vector p9-Dao-FLPi. The line C44.4.146 was shown to carry only the cisgene *FB_MR5*, controlled by its native regulatory sequences and no transgenes were detected by PCR or Southern blot following heat induced recombinase-mediated elimination of the selectable markers. Although this line contains up to 452 bp of vector sequences, it still matches the original definition of cisgenesis. A single insertion of T-DNA into the genome of 'Gala Galaxy' in chromosome 16 was identified. Transcription of *FB_MR5* in line C44.4.146 was similar to the transcription in classically bred descendants of *Mr*5. Three independent shoot inoculation experiments with a *Mr*5 avirulent strain of *Erwinia amylovora* were performed using scissors or syringe. Significantly lower disease symptoms were detected on shoots of the cisgenic line compared to those of untransformed 'Gala Galaxy'. Despite the fact that the pathogen can overcome this resistance by a single nucleotide mutation, this is, to our knowledge, the first prototype of a cisgenic apple with increased resistance to fire blight.

## Introduction

Apple is one of the most important fruit crops worldwide considering its production level of 80.8 million tons per year [1]. Apple production relies on a small number of commercial cultivars. Most of today’s successful cultivars such as 'Braeburn', 'Fuji', 'Gala', 'Golden Delicious', 'Jonagold' and 'Cripps Pink' are susceptible to fire blight [2, 3] and to other major plant diseases like apple scab. For a long time resistance to diseases was often neglected in many breeding programs as the main focus was on fruit quality combined with optimal agronomical properties [4] and only in the last decades of the previous century, did disease resistance begin to gain relevance [5–9]. This occurred with increasing awareness of the ecological and economical costs of disease management strategies relying on plant protective chemicals only.

High levels of resistance to diseases are often found in wild *Malus* accessions, e.g. the *Rvi6* (alias *Vf*) resistance gene from *M*. *floribunda* clone 821 conferring resistance to apple scab [10] caused by *Venturia inaequalis*, or the *FB_MR5* of *M*. ×*robusta* 5 which confers resistance to fire blight [11] caused by *Erwinia amylovora*. When such resistances are introduced into a breeding program, at least 5 pseudo backcrosses are then necessary to remove the unwanted properties inherited from the wild ancestor (e.g. small fruits) together with the resistance. Considering that the juvenile phase of the domesticated apple lasts between three and twelve years (and linkage drag), the introduction of a resistance from a wild source takes between 20 to 50 years until a cultivar with fruit of marketable quality can be released [12].

The rapid advancement in gene cloning in apple and in genetic transformation methods allows the deployment of resistances by means of genetic modification [13]. In particular cisgenesis may be considered for the goal of adding a particular resistance gene to cultivars of commercial value [14]. Schouten et al. [14] defined a cisgenic plant as a plant that does not contain any transgenes and that has been genetically modified with one or more genes (containing introns and flanking regions such as native promoter and terminator regions in sense orientation), isolated from a crossable donor plant. The recent identification and cloning of several apple resistance genes like *Rvi6* [15], *Rvi15* [16, 17], *Pl2* [18], *FB_MR5* [19], as well as the development of methods to generate marker-free genetically modified apple plants [20–22] are the basic requirements for developing cisgenic apple lines. The first cisgenic apple lines with scab resistance were developed by Vanblaere et al. [23]. Würdig et al. [24] and Krens et al. [25] later developed further cisgenic apple lines with *Rvi6*.

The most problematic bacterial disease in apple orchards is fire blight, caused by the Gram-negative bacterium *Erwinia amylovora* (Burrill) Winslow et al. [26]. It causes severe losses in years of heavy epidemics, with economical costs reaching up to several million Euros per year in single European countries or US states [27–29].

The use of fire blight resistant cultivars is a tool that is currently not popular in the management of the disease. This is because the very few commercial cultivars showing high levels of resistance to the disease including ‘Rewena’ [30] and ‘Enterprise’ [31], do not fully meet retailers and consumers demand, because their level of fruit and tree quality is not comparable with the top world varieties. Good levels of fire blight resistance were found in individual accessions of different wild apple species like *M*. *fusca* [32], *M*. *baccata* [33], *M*. *prunifolia* [34], *M*. ×*atrosanguinea* [35], *M*. ×*robusta* var. *persicifolia* [36] and *M*. *sieversii* [37] as well as in ‘Evereste’ [38, 39], *M*. *floribunda* 821 [38] and *Malus* ×*robusta* 5 [11].

In the last decade the resistance of the crab apple genotype *Malus* ×*robusta* 5 has been studied in depth. Peil et al. [11] identified a major QTL on linkage group 3 of *M*. ×*robusta 5*. Later on, Fahrentrapp et al. [40] identified a candidate resistance gene in this region, which was designated as *FB_MR5*, and predicted to code for a CC-NBS-LRR resistance protein. The functionality of the *FB_MR5* gene under its native promotor and terminator sequences and under the constitutive CaMV 35S promoter and ocs terminator, respectively, was demonstrated [19]. Vogt et al. [41] suggested that this resistance undergoes a gene-for-gene interaction, as a single amino acid substitution in the AvrRpt2_EA_ gene of *E*. *amylovora*, is sufficient to allow the pathogen to become virulent and to overcome this resistance.

In the present study the generation of a cisgenic 'Gala Galaxy' line carrying the *FB_MR5* gene showing increased resistance to fire blight is presented. *In vitro* shoot cultures of 'Gala Galaxy' were transformed with the novel binary transformation vector p9-Dao-FLPi [24], allowing the post-transformation heat induced flippase (*Flp*) based removal of the excisable cassette containing three transgenes (*NptII*, *Flp* and *dao1*). Although it was not possible to use the selectable marker *dao1* to increase the number of regenerants of lines without transgenes, a cisgenic line C44.4.146 was identified. Being *FB_MR5* controlled by its native promotor and terminator, this line matches the definition of cisgenic plants of Schouten et al. [14]. The level of fire blight resistance of this line was assessed by means of two different shoot inoculation methods and compared with untransformed 'Gala Galaxy' plants. Further characterization of this line involved the assessment of the number of T-DNA integrations, site of integration in the genome, and transcription level of the *FB_MR5* gene in this line compared to conventionally bred genotypes carrying the *FB_MR5* gene. To our knowledge, this is the first report of a cisgenic apple with increased resistance to fire blight.

## Results

### Generation of the cisgenic line C44.4.146

**Fig 1:**
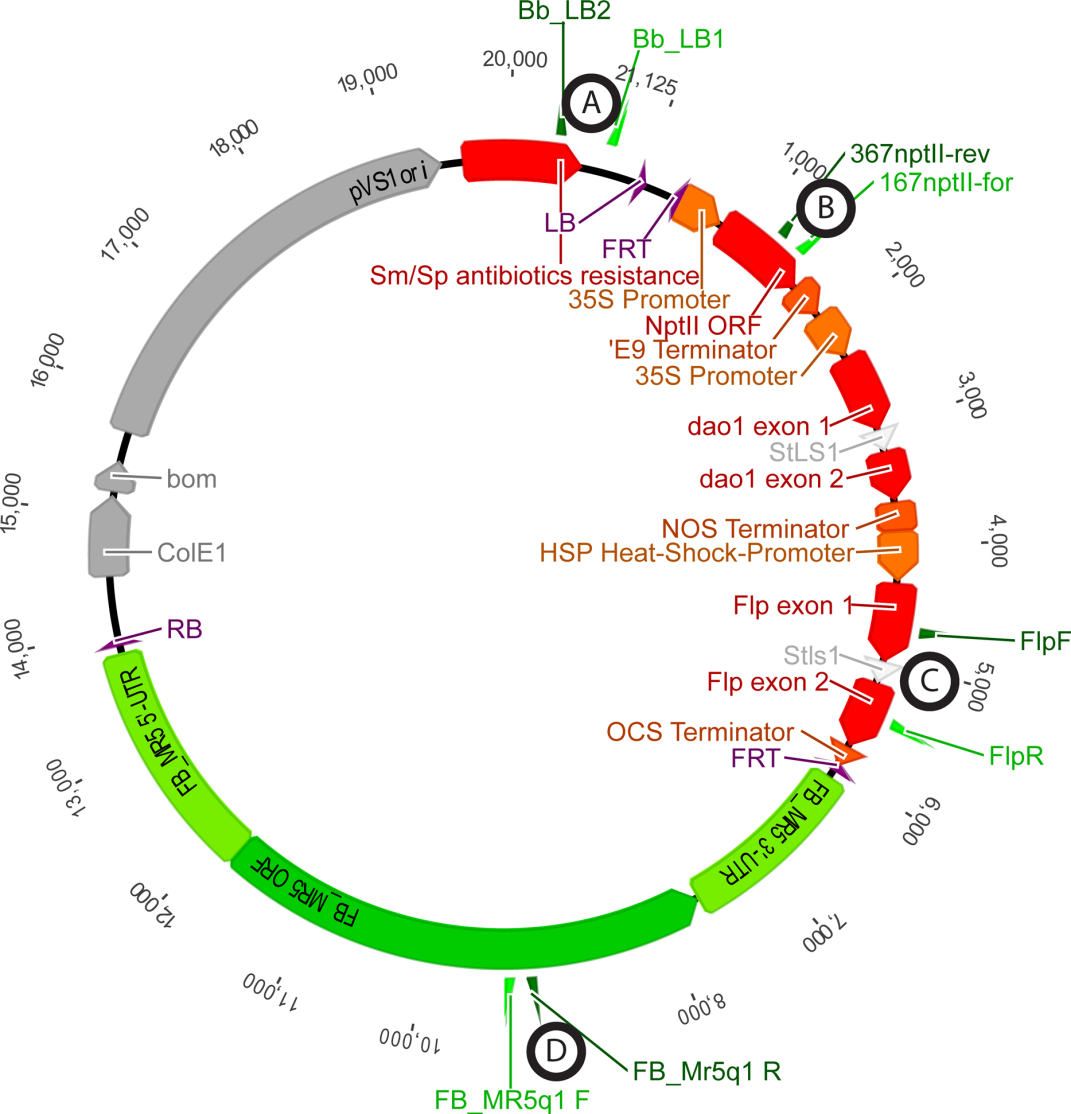
Map of the p9-Dao-FLPi-FB_MR5 vector used for transformations. Apple exogenous genes (transgenes) are colored in red, cisgene *FB_MR5* with native coding elements in green and vector backbone in grey. Left border (LB), flippase recognition target (FRT), *neomycin phosphotransferase II* (*NptII*), *D-amino acid oxidase 1* (*dao1*), *flippase* (*Flp*) and right border (RB) are indicated. Primers are shown inside the vector as triangles. Relative position of amplicons A, B, C and D for further analysis (Figs 2 and 3) are indicated. Amplification of A indicates the presence of sequences beyond the left border.

**Fig 2:**
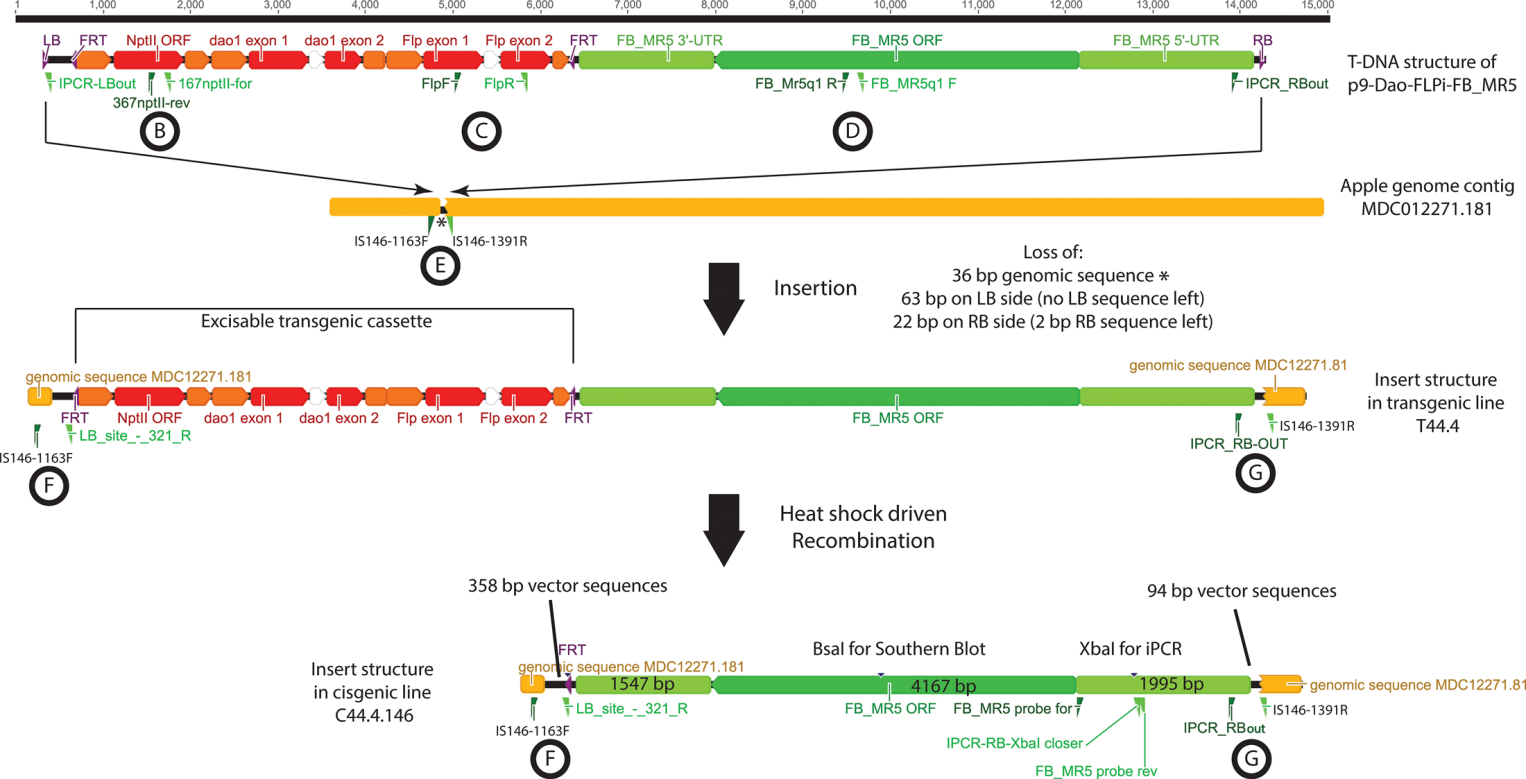
Schematic representation of insertion site before and after heat shock induction leading to cisgenic line C44.4.146. Transgenes *neomycin phosphotransferase II* (*NptII*), *D-amino acid oxidase 1* (*dao1*) and *flippase* (*Flp*) are shown in red, genomic DNA of 'Golden Delicious' in yellow, regulatory elements in orange and *FB _MR5* and regulatory sequences in green. Vector sequences in the cisgenic line are colored black and flippase recognition target (FRT) and original border sites are indicated in purple. Circles with letters indicate the amplicons resulting by PCR with the corresponding primers. Amplification of B and C indicates presence of the excisable cassette, amplification of D the presence of the cisgene *FB_MR5*. Amplicon E flanks the insertion site. F and G amplify the junction T- and genomic DNA at LB, and RB, respectively. In the structure of the insert in C44.4.146 both restriction sites *Bsa*I and *Xba*I, used for Southern blot analysis and iPCR, respectively, are indicated.

**Fig 3:**
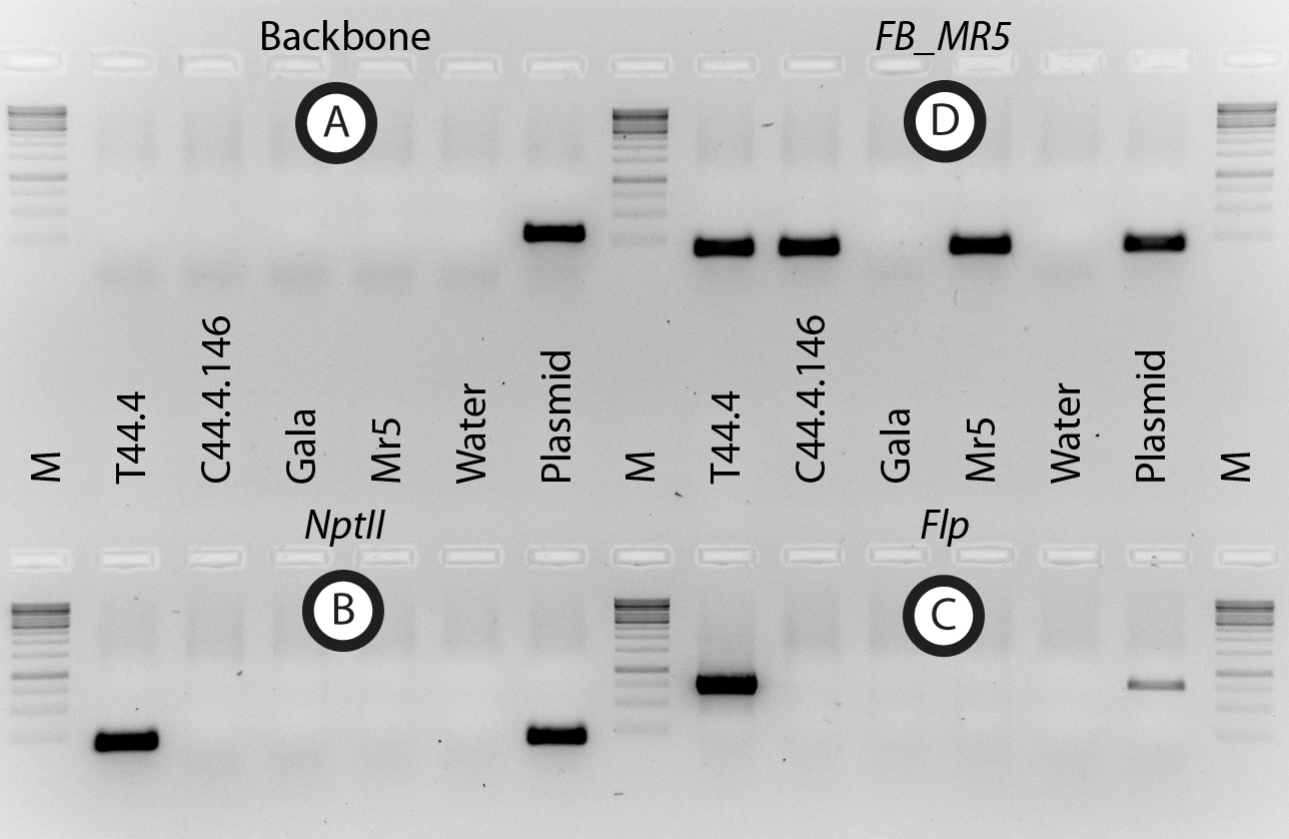
Results of PCR tests to verify the presence / absence of backbone, *FB_MR5*, *NptII* and *Flp*. DNA template used for PCR was always loaded in the following order: Transgenic motherline T44.4, cisgenic line C44.4.146, 'Gala Galaxy' *in vitro* (Gala), *Malus* ×*robusta* 5 (Mr5), negative control (water) and vector p9-Dao-FLPi-FB_MR5 (plasmid). Marker (M) is GeneRuler^™^ 1 kb DNA ladder (Thermo Fisher scientific Inc. ©, Waltham, USA). Location of the backbone amplicon A is shown in Fig 1. Positions of primers of amplicons B, C and D are shown in Figs 1 and 2.

From two Agrobacterium-mediated transformation experiments T44 and T45, with vector p9-Dao-FLPi-FB_MR5 (Fig 1), eleven and two transgenic lines were obtained, corresponding to transformation efficiencies (transformants per explants) of 13.8% and 0.7%, respectively. Integration of *FB_MR5* (amplicon D, Fig 2) and *NptII* (amplicon B, Fig 2) was confirmed in all 13 lines and in two cases of T44, integration of backbone sequences was detected (S1 Fig). Those two lines were removed, as lines with backbone usually do not lead to cisgenic plants. Nine out of the eleven remaining transgenic lines (seven from T44 and two from T45) produced sufficient leaf material to be subjected to heat shock. About 3,200 explants were subjected to heat shock activation of the *Flp* recombinase. The negative D-amino acid / *dao1* selection system proposed by Hättasch et al. [42] could not be applied, as it was recently found that the selective medium containing D-Ile hinders the regeneration [24]. On this account PCR was used to screen the obtained putative cisgenic regenerants. Four out of 447 regenerants contained *FB_MR5* and were free of selection marker (S2 Fig). Because all four regenerants originated from calli from the same transgenic mother line T44.4, and assuming a consistent excision process, we consider them to be the same genotype. Nevertheless only one shoot of the four regenerants was designated as line C44.4.146 and used for micrografting on 'Golden Delicious' seedlings for further characterization. PCR confirmed the presence of *FB_MR5* (amplicon D, Fig 3), absence of both transgenes *NptII* (amplicon B, Fig 3) and *Flp* (amplicon C, Fig 3) plus absence of backbone sequences beyond the left border in this line after micrografting (amplicon A, Fig 1).

### Copy number of C44.4.146

**Fig 4:**
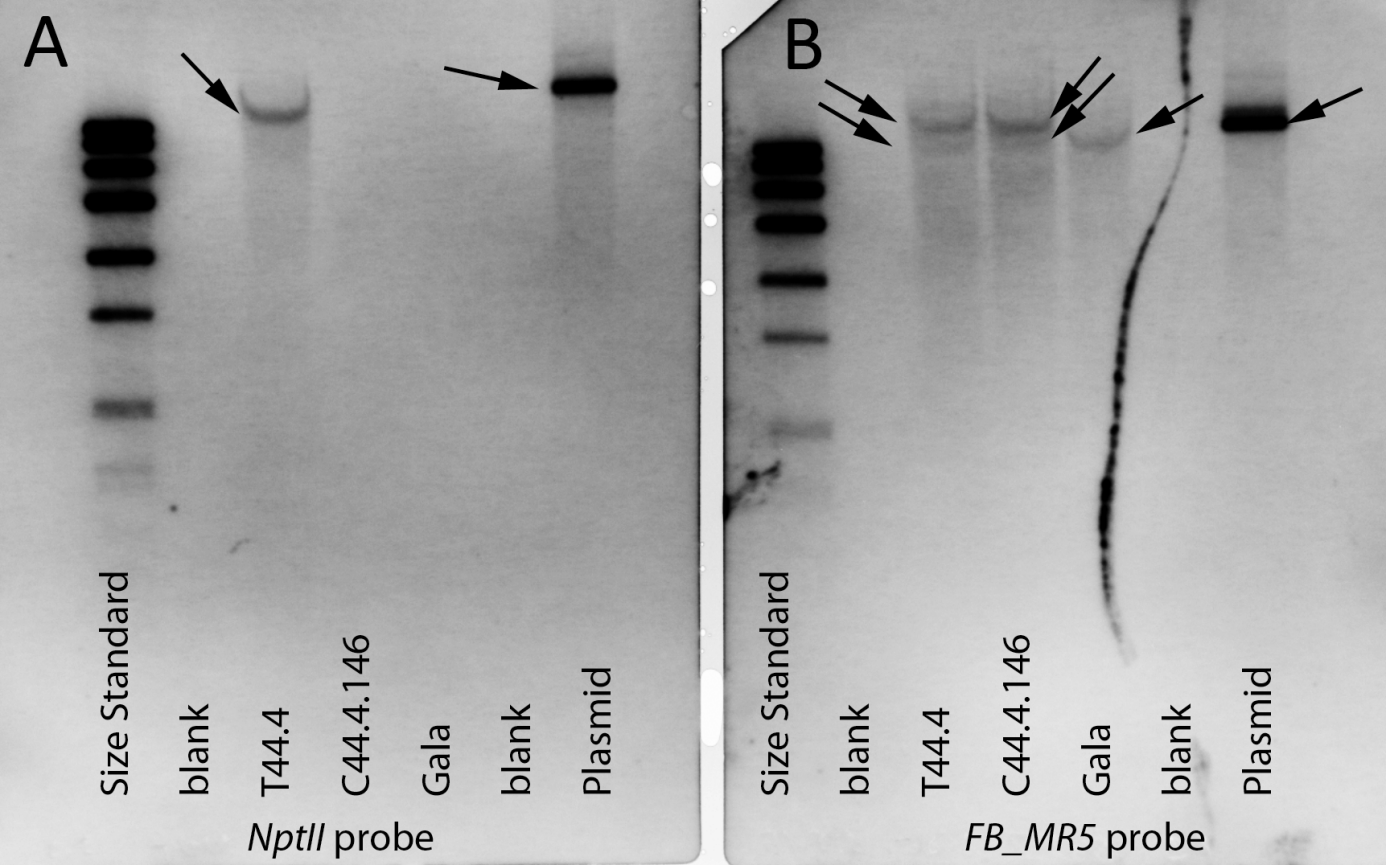
Determination of copy number by Southern hybridization using a *NptII* (A) or *FB_MR5* (B) specific probe. A) A single copy of *NptII* was detectable in the transgenic motherline (T44.4) and in the vector p9-Dao-FLPi-FB_MR5 (plasmid) when probed with an *NptII* specific digoxygenin (DIG)-labelled probe. B) Using the *FB_MR5* probe an unspecific band was detected in 'Gala Galaxy' *in vitro* (Gala), line C44.4.146 and its motherline T44.4. An additional, *FB_MR5* specific band was visible in T44.4, C44.4.146 and in p9-Dao-FLPi-FB_MR5.

Using Southern hybridization a single specific band was detected with an *NptII* probe in the transgenic motherline T44.4, indicating that originally one copy of the T-DNA had been inserted (Fig 4A). In C44.4.146 this band was not detected confirming excision of the excisable cassette (Fig 4A). As expected 'Gala Galaxy' showed no hybridization with this probe. Southern hybridization with an *FB_MR5* probe allowed the identification of an unspecific band in 'Gala Galaxy', while C44.4.146 and T44.4 showed a single additional *FB_MR5*-specific band (Fig 4B).

### Integration site of C44.4.146

**Fig 5:**
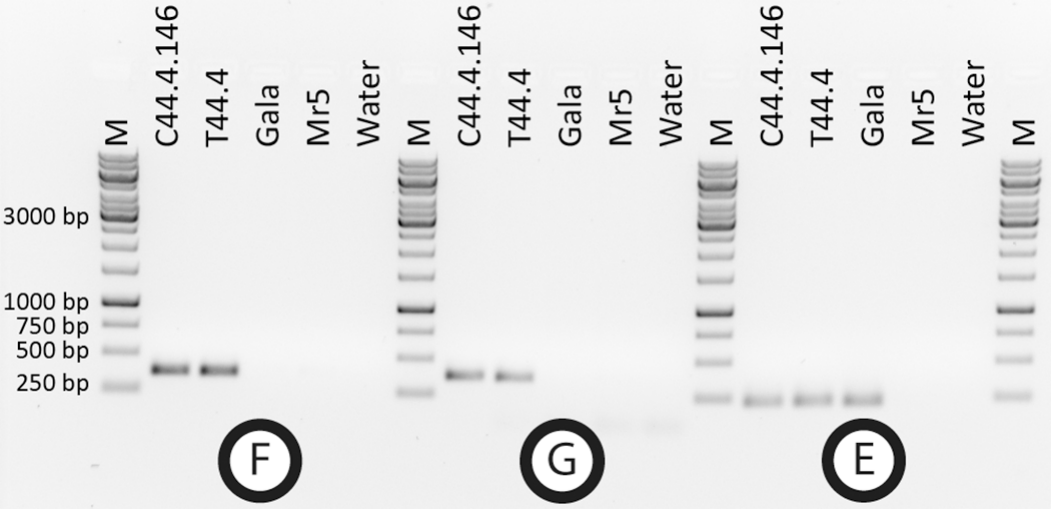
Results of PCR test of amplicons spanning the insertion site. PCR was performed with DNA of cisgenic line C44.4.146, T44.4, ‘Gala Galaxy’ *in vitro* (Gala) and the resistance donor *M* x*robusta* 5 (Mr5). Water was used as negative control. Amplicons F and G are specific to T44.4 and C44.4.146 and can be used for discrimination purposes of these lines from ‘Gala Galaxy’. Location of the amplicons and corresponding primers are shown in Fig 2. It is presumed that E amplifies in 'Gala Galaxy' two alleles while only one is amplified in T44.4 and C44.4.146 (Fig 2 and results) as the second amplicon, containing the inserted T-DNA sequence, is too large to be amplified.

The genomic region flanking the insertion site in line C44.4.146 was identified using iPCR. The sequence from the RB site showed highest level of sequence identity to a sequence of the 'Golden Delicious' contig MDC012271.181 located on chromosome 16. No gene was predicted at the insertion site. Furthermore, mapping the reads generated by Gusberti et al. [43] did not reveal any novel transcript at the insertion site. The closest gene was MDP0000141330 (coding for a transmembrane transporter) at about 600 bp. Primer pairs IS146-1163F/IS146-321R (amplicon F, Fig 5), and IS146-1391R/iPCR-RBout (amplicon G, Fig 5) were designed to characterize the insertion site in line C44.4.146 at left border (LB) and right border (RB), respectively (Fig 2). These primers can be used to discriminate between C44.4.146 or T44.4 lines and the not genetically modified ‘Gala Galaxy’. Using the primers IS146-1163F/IS146-1391R (amplicon E, Fig 2), which flank the insertion site, no large fragments (eight kb in C44.4.146 or 14 kb in T44.4) were amplified by standard PCR conditions but a small PCR product was amplified corresponding to the second allele of contig MDC012271.181 present in C44.4.146, T44.4 and presumably in both alleles of 'Gala Galaxy' (amplicon E, Fig 5).

Sequencing of the F and G PCR products (Fig 2) from C44.4.146 covering the genomic T-DNA junctions (amplicons F and G, Fig 2) revealed that 22 base pairs (bps) of the RB sequence were trimmed away and only two bps of the RB were found in C44.4.146, while a total of 63 bps including the LB itself were trimmed away on the opposite T-DNA side (Fig 2). The T-DNA insertion led to the loss of 36 bps of apple genomic sequences (MDC012271.181, bps 1287–1322). In addition, in line C44.4.146, beside the endogenous sequences of *FB_MR5* gene (4167 bps) flanked by 1995 bp native 5’-UTR and 1547 bp native 3’-UTR, 358 bps (including flippase recognition target (FRT)) and 94 bps of p9-Dao-FLPi vector derived apple exogenous sequences were found at LB side and RB side of the T-DNA, respectively.

### Transcription of *FB_MR5*

**Fig 6:**
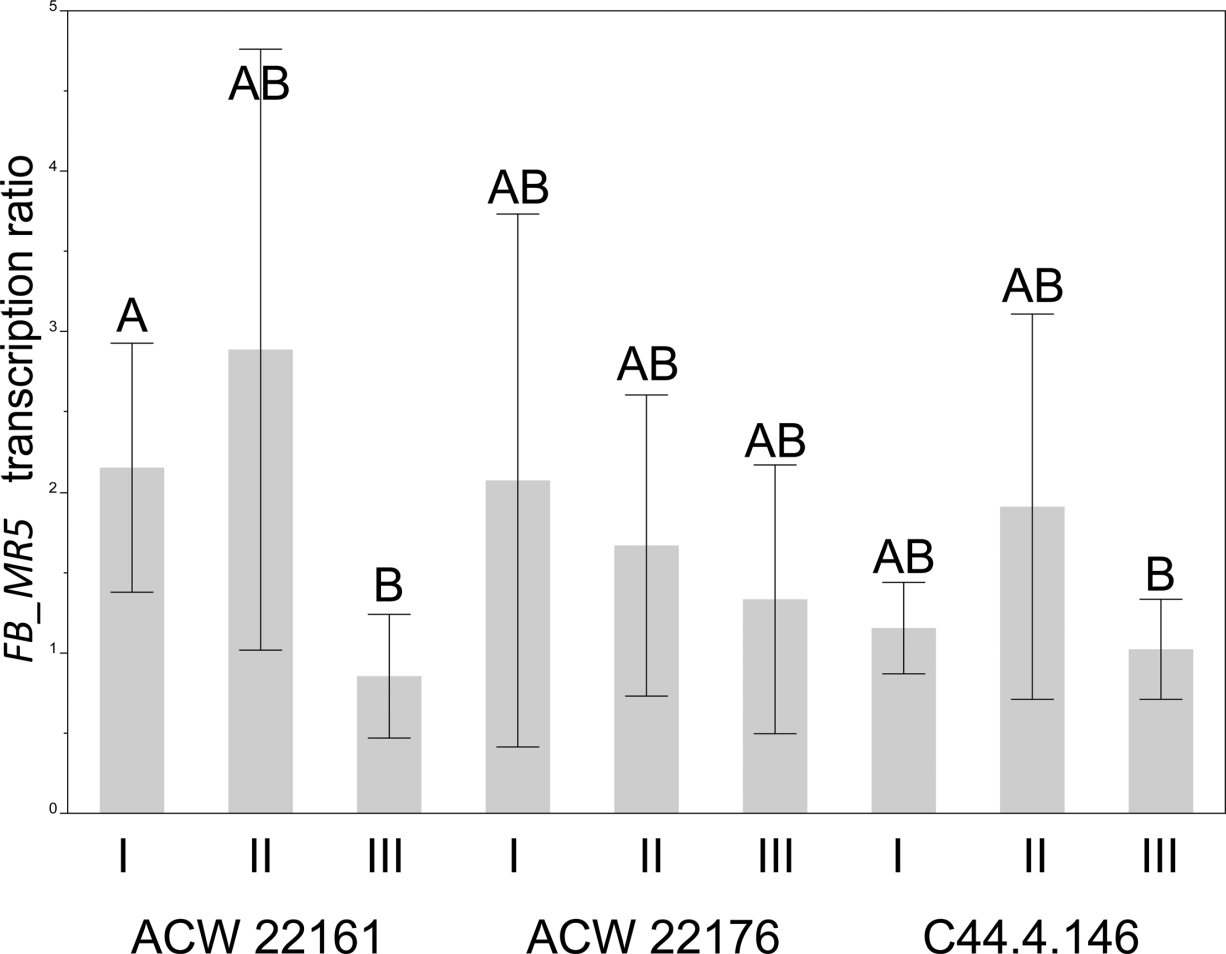
Comparison of the *FB_MR5* transcription level of C44.4.146 and two classically bred *FB_MR5* lines (ACW 22161 and ACW 22176). Three biological replicates are shown (I, II and III). For each of them three to eight technical replicates were created. Different capital letters indicate statistically significant differences (Steel-Dwass test with α = 0.05).

Transcription level of *FB_MR5* was estimated and compared with two classically bred *FB_MR5* accessions (ACW 22161 and ACW 22176). It was observed that the CT values of both target amplicons, *EF1*α and *FB_MR5*, differed largely, with CT values for *EF1*α in the range between 20 and 28, while for *FB_MR5* they were between 30 and 50, whereas only few samples showed CT_FB_MR5_ values above 40 (S1 Table). However we observed no significant difference between transcription ratio in line C44.4.146 and in accession ACW 22176 and only one replicate of ACW 22161 (I) differed significantly from one replicate of C44.4.146 (III, Fig 6). This replicate of ACW 22161 (I) also differed significantly from its own biological replicate (III, Fig 6). As a negative control the fire blight susceptible 'Gala Galaxy' was used. No amplification of the *FB_MR5* probe was observed (S1 Table).

### Fire blight resistance of the cisgenic line C44.4.146

**Fig 7:**
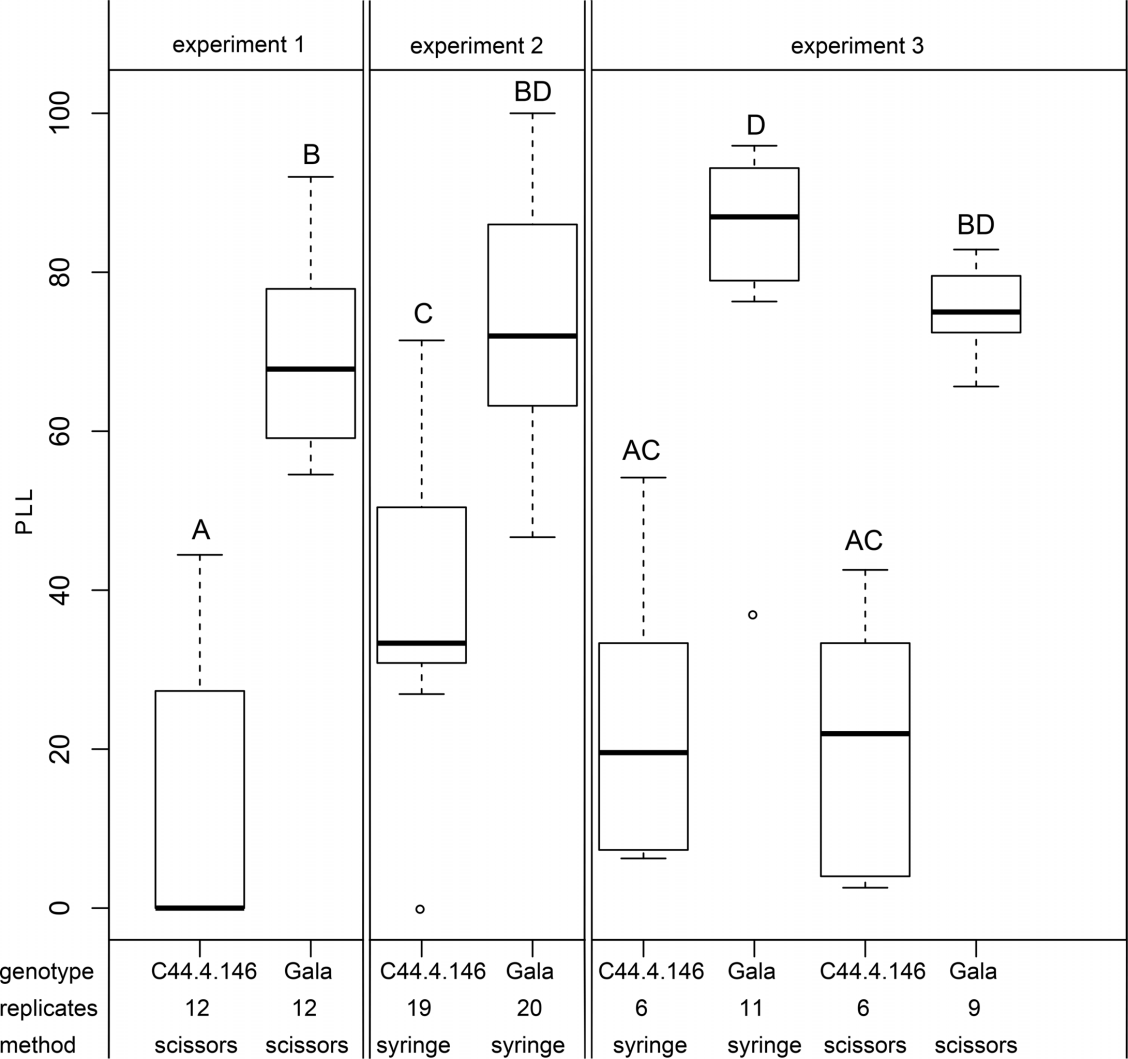
Boxplot showing fire blight severity between greenhouse shoots of both C44.4.146 and 'Gala Galaxy' from *in vitro* culture. Disease severity is expressed in percentage of lesion length (PLL) of the shoot of cisgenic (C44.4.146) and 'Gala Galaxy' *in vitro* plants (Gala), 21 days after inoculation in three independent experiments. Number of inoculated plants is indicated (replicates). Inoculations were performed either using scissors or syringe method. Each small box delimits values between 25% and 75% of the group. Bold horizontal line represents median of group. Whiskers are drawn for obtained values that differ least from median ± 1.5 interquartile ranges. Different letters show statistically significant differences (Steel Dwass test with α = 0.05) between groups in all experiments. Outliers are shown as empty circles.

**Fig 8:**
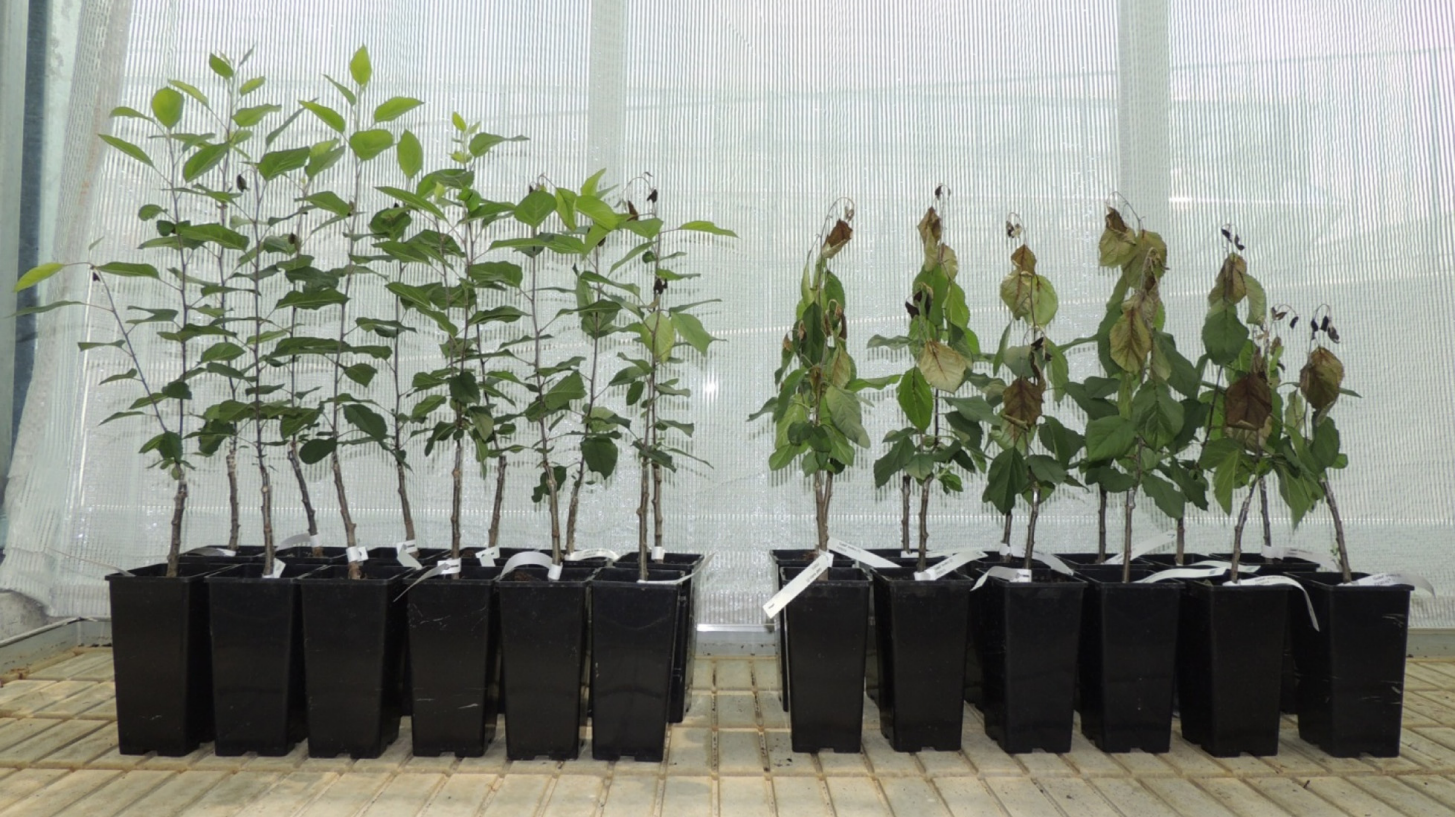
Results of fire blight inoculation on grafted apple line C44.4.146 (left) and 'Gala Galaxy' *in vitro* (right). Picture taken 20 days after scissors inoculation with Ea222_JKI (Fig 7, experiment 1).

The cisgenic line C44.4.146 showed increased fire blight resistance compared to non-transformed 'Gala Galaxy' control plants in three independent inoculation experiments 21 days after inoculation with *E*. *amylovora* strain Ea222_JKI in the greenhouse (Fig 7). In the first experiment the cisgenic line C44.4.146 showed significantly (Wilcoxon test p-value < 0.0001) less symptoms (11.4% ± 17.7% PLL) compared to 'Gala Galaxy' (69.3% ± 12.1) three weeks after scissors inoculation. Four out of twelve inoculated shoots of C44.4.146 showed small necroses at the stem, while the other eight shoots showed necroses limited to the midvein of the inoculated leaves. In contrast, all 'Gala Galaxy' plants showed strong stem necroses (Fig 8). In a second inoculation experiment when shoots were directly injected with *E*. *amylovora* using a syringe, the cisgenic line C44.4.146 showed a mean PLL of 41.1% ± 15.0%, which resulted to be significantly different from the mean PLL of 'Gala Galaxy' showing 73.9% ± 16.5% (Wilcoxon test p-value < 0.0001, Fig 7). All plants of both genotypes except one plant of C44.4.146 showed necrosis expanding along the stem. In a third experiment both inoculation methods were compared simultaneously. 21 days after inoculation, all inoculated plants showed some necroses. The cisgenic C44.4.146 line showed 23.4% ± 19.3% PLL while 'Gala Galaxy' showed 87.9% ± 7.2% PLL after syringe inoculation (Fig 7). Similar results were observed for the cisgenic plants (21.1% ± 16.6% PLL) and 'Gala Galaxy' (75.2% ± 5.5% PLL) when inoculated using scissors (Fig 7). Using the Steel Dwass test, we observed significant differences (p-value < 0.02) between the PLL of line C44.4.146 and 'Gala Galaxy', independent of the inoculation method used (experiment 3, Fig 7).

## Discussion

In the present study a cisgenic apple of the cultivar 'Gala Galaxy' with increased resistance to fire blight was developed. The susceptible cultivar 'Gala Galaxy' acquired fire blight resistance by insertion of the resistance gene *FB_MR5* flanked by its 1995 bp native 5’-UTR and 1547 bp native 3’-UTR. The same regulatory sequences were shown to effectively regulate the function of *FB_MR5* in transgenic lines developed by Broggini et al. [19]. Cisgenic transformation was achieved using a novel vector p9-Dao-FLPi [24] for *Agrobacterium*-mediated transformation allowing the post-transformation heat-induced *Flp*-based excision of the transgene cassette. As a negative selection marker to exclude cells in which recombination did not occur, the gene *dao1*, encoding *D-aminooxidase 1* [42], is present in the excisable cassette of p9-Dao-FLPi. However, this negative selection marker could not be applied, as it has recently been shown that the use of D-Ile containing medium (to remove cells in which the recombination did not occur) completely hinders the formation of cisgenic shoots and moreover transgenic shoots survived for several months on it [24]. Therefore we decided to regenerate lines without application of the D-Ile / *dao1* system, using a regeneration medium without D-Ile and selecting the regenerants by PCR. Thereby cisgenic regenerants can be identified as they result in no PCR product after amplification with primers for the selectable marker cassette, in contrast to transgenic regenerants. Chimeric cisgenic-transgenic plants should lead to amplification of *NptII* and *Flp* according to the detection limit of PCR. Despite the hindering circumstances, a cisgenic genotype was identified (Fig 3). Nevertheless, a transformation vector with an effective negative selection would have been of great help to increase the number of regenerating cisgenic genotypes. Substitution of *dao1* in p9-Dao-FLPi with *codA* could result in a pMF1-alternative vector with functional negative selection. This strategy was also proposed by Würdig et al. [24]. An approach with an alternative selectable marker could be the use of the *MdMYB10* gene [44] originating from the apple gene pool, leading to visually detectable, red colored calli, shoots and fruits [25].

Line C44.4.146 was regenerated and molecularly investigated. We consider C44.4.146 as a cisgenic line, as PCR revealed that C44.4.146 amplified *FB_MR5* but no transgenic selection markers (Fig 3). Southern blot hybridization indicated a single T-DNA insertion in this genotype, as well as the absence of *NptII* in the final cisgenic line C44.4.146 (Fig 4). On this account we consider the cisgenic line C44.4.146 to carry a single T-DNA insertion. *FB_MR5* transcription analysis revealed that the transcription in this line was not different from the transcription in the *FB_MR5* accessions ACW 22161 and ACW 22176 (Fig 6). Cisgene insertion in C44.4.146 occurred as a single copy on chromosome 16 (Fig 4). Gene prediction on contig MDC012271.181 indicates that no apple endogenous gene has been disrupted by the T-DNA insertion in line C44.4.146. 85 bps of T-DNA ends were trimmed away (63 bps on LB side, 22 bps on RB side) and T-DNA insertion resulted in the deletion of 36 bps genomic sequences (Fig 2). Trimming of T-DNA ends was also observed in cisgenic apple developed by Vanblaere et al. [45]. The extent of foreign sequences resulting in the final product generated using p9-Dao-FLPi without this trimming is larger than using pMF1 (538 bps vs 140 bps, [20]). While designing novel vectors to generate cisgenic genotypes only foreign sequences shorter than 20 bps can be present in the final product [46] to match the EFSA definition of cisgenic crops used to formulate their safety assessment on cisgenesis [47]. However, a product matching this definition can hardly be achieved when using recombinase recognition sequences (of which one copy remains in the final cisgenic product) that are at least 34 bps long for the *Flp* / FRT system [48] in p9-Dao-FLPi and 58 bps long for the R / Rs system in pMF1 [20]).

Three different inoculation experiments, using scissors and/or syringe shoot inoculation, showed consistently that line C44.4.146 is significantly more fire blight resistant than the wild type (Figs 7 and 8). The comparison of the results of experiments 1 and 2 seems to indicate that while inoculating using a syringe, more severe fire blight symptoms are induced in the cisgenic line. However, when C44.4.146 and ‘Gala Galaxy’ plants were inoculated with the two inoculation methods in the same experiment (experiment 3), this effect was no longer observed (Fig 7). Flower susceptibility is very important for controlling the fire blight disease, as most of the infections occur during flowering. Resistance of this cisgenic line upon inoculation of the flowers with the pathogen has still to be investigated.

We discourage the single use of the introduced fire blight resistance gene *FB_MR5* by conventional breeding or by cisgenesis for cultivation, as it is known that a single point mutation leading to amino acid substitution in the pathogen can result in a virulent strain [41]. *FB_MR5* virulent strains of *E*. *amylovora* have already been identified in North America, while such strains have not yet been identified in Europe. The mutation rate of the codon 43 switch of rpsL gene from K to R altering the ribosomal protein S12 leading to streptomycin resistance in most of the strains of *E*. *amylovora* was estimated to 4 x 10^−9^ per bacterial generation [49]. If we assume the same mutation rate for the switch from C-Allele to the S-Allele of AvrRpt2_EA_ (leading to *FB_MR5* virulent strains) and we consider that *E*. *amylovora* populations ranging from 10^3^ to 10^7^ cfu have been detected on stigmas of flowers of different rosaceous hosts after natural infection [50, 51], it can be assumed that *Mr5*-virulent *E*. *amylovora* strains are already present in Europe and can rise very quickly once we start selecting for them (i. e. by planting *FB_MR5* genotypes at commercial scale). Therefore it can be anticipated that if used alone, this resistance would not be durable. Whatever use (e.g. classical breeding, transgenesis or cisgenesis) is made of *FB_MR5*, this resistance must be further combined with other (vertical or horizontal) resistances as suggested by McDonald and Linde [52] and Emeriewen et al. [53].

## Experimental Procedures

### Generation of the cisgenic line C44.4.146

Binary vector p9-Dao-FLPi-FB_MR5 (Fig 1) was constructed by a DNA cloning company (DNA Cloning Service, Hamburg, Germany) and transformed into *Agrobacterium tumefaciens* strain GV3101pMP90RK. This vector was derived from P9 and was designed to carry on the T-DNA an excisable cassette flanked by FRT recombination sites. This excisable cassette contains the *NptII* kanamycin resistance selection gene for plant selection, the *D-amino oxidase 1* gene (*dao1*), originally planned to be used as negative selection marker, and the *Flp* recombinase under control of a heat shock promoter [42]. The same vector carrying *HcrVf2* was recently published by Würdig et al. [24]. Instead of *HcrVf2* and its native regulatory elements, the *FB_MR5* gene (4167 bps) flanked by its 1995 bps native 5’-UTR and 1547 bps native 3’-UTR was cloned, using the same primers and restrictions sites as described by Broggini et al. [19] for generating vector 390p95N-Mr5FB1.

Transgenic plants were regenerated by transforming *in vitro* explants of the cultivar 'Gala Galaxy' following the protocol as described by Szankowski et al. [54] and Vanblaere et al. [23]. Young leaves of the *in vitro* plants were cut in explants (a total of 80 in T44 and 300 in T45), co-cultured with the transformed agrobacteria carrying the vector p9-Dao-FLPi-FB_MR5 and regenerated on a medium containing ticarcilin and kanamycin to select for pure (agrobacteria-free), transformed, transgenic calli. Once the plants started to regenerate they were transferred to elongation medium [23] and propagated every four to six weeks. To test if the shoots were successfully transformed and thus to confirm integration of *FB_MR5* and *NptII*, all 13 lines growing on selective medium containing kanamycin were investigated by PCR with the primer pairs FB_MR5q1 F/FB_MR5q1 R (amplicon D, Fig 2) for *FB_MR5* and 167nptII-for/367nptII-rev (amplicon B, Fig 2) for *NptII*. In order to identify lines that contain backbone sequences beyond the left border, PCR using primer pair Bb_LB1/Bb_LB2 (Amplicon A, Fig 1) was performed.

As soon as sufficient differentiated shoots were available, about 3,200 explants of nine transgenic lines were subjected to heat shock in an incubator (four hours at 42°C on regeneration medium without kanamycin) to activate excision of the trangenic cassette containing the selectable marker genes *NptII*, *dao1* and the *Flp* recombinase gene as well. Following heat treatment explants were cultured on regeneration medium without kanamycin for two weeks in the dark. After this period, explants were put on non-selective elongation medium, like during the transformation procedure, and started to form calli. Elongating shoots were transferred to elongation medium as soon as shoots were detectable or cells started to differentiate. To detect the cisgenic regenerants, they were screened for absence of *NptII* and *Flp* and for presence of *FB_MR5* by PCR using the method of Frey et al. [55]. For this purpose primers 167nptII-for/367nptII-rev [54], FlpF/FlpR [22] and FB_MR5q1 F/FB_MR5q1 R [56] were used (Table 1 and Fig 2).

**Table 1:** List of the primers used in this study.

Primer name	Sequence 5’-3’	Reference	Amplicon^a^
Bb_LB1	CGCAATAGTTGGCGAAGTAATCGC	This study	A
Bb_LB2	GGTGGAGCTTGCATGTTGGTTTC	This study	A
FB_MR5q1 F	TTTATGGAGAGTGCTCCTTGC	[56]	D
FB_MR5q1 R	AGCGAATCAAGGTTCTCTGG	[56]	D
FbMr5_SondeF	TCACAACTAATCACAGCTGCA	This study	
FbMr5_SondeR	AACATTATGATCCCACCCTACG	This study	
FlpF	CATCGGAAGAAGCAGATAAGGG	[22]	C
FlpR	TCAACTCCGTTAGGCCCTTCAT	[22]	C
iPCR-RBout	ATCTCACGTCATCCATGCCC	This study	G
iPCR-RB-XbaI-closer	TCGTAGCAAATTGAAGAGGTCT	This study	
IS146-1163F	TCGTTTTCAGTTCGATATGAGCAAG	This study	E, F
IS146-1391R	ACATGCATAATGGTGATGAGGG	This study	E, G
IS146-LB-321R	GCTAGAGCCGATCGTGAAGT	This study	F
nptII_F	ACAAGATGGATTGCACGCAGG	[19]	
nptII_R	AACTCGTCAAGAAGGCGATAG	[19]	
167nptII-for	CCACAGTCGATGAATCCAGA	[54]	B
367nptII-rev	AGCACGTACTCGGATGGAAG	[54]	B

^a^ The relative positions of the primers on the sequence of T44.4 are shown in Figs 1 and 2.

A shoot of C44.4.146 and non-transformed 'Gala Galaxy' control plants, which simultaneously experienced *in vitro* culturing were then micrografted on 'Golden Delicious' seedlings following the procedure described by Joshi [57] and acclimatized to greenhouse conditions. This latter genotype is described in this manuscript as “'Gala Galaxy' *in vitro*”. DNA of the micrografted C44.4.146 plant, its motherline T44.4, *Malus* ×*robusta* 5 and 'Gala Galaxy' *in vitro*, was retested by PCR for absence of sequences beyond the left border, presence of *FB_MR5* and absence of the selectable marker genes N*ptII* and *Flp* using the corresponding primers (Figs 2 and 3).

### Fire blight resistance test of the cisgenic line C44.4.146

Between six and twenty plants of each genotype grafted on M9T337, were subjected to fire blight resistance tests with *E*. *amylovora* strain EA222_JKI in each of the three independent experiments performed. Only actively growing plants that reached at least a shoot length of 13.0 cm were considered. Two inoculation methods were used: scissor inoculations (experiments 1 and 3) were performed as described by Peil et al. [11] and syringe inoculation (experiments 2 and 3) as described by Khan et al. [58]. In all three experiments (experiments 1–3) an *E*. *amylovora* suspension in phosphate buffered saline with an optical density (OD_600_) adjusted to about 1.0 (1.06, 0.99, 1.09, respectively), corresponding to about 10^9^ cfu / ml, was used. The percentage of lesion length (PLL) was recorded 21 days post inoculation.

### Copy number of C44.4.146

Southern hybridization was performed as described by Broggini et al. [19] with minor changes. DNA (10 μg) from each line and plasmid p9-Dao-FLPi-FB_MR5 was digested with 100 units *Bsa*I (Thermo Fisher Scientific Inc. ©, Waltham, USA). Cleaved DNA was separated on a 0.8% agarose gel and transferred onto a nylon membrane (Roche Diagnostics, Mannheim, Germany). A DIG-labelled *NptII* probe was amplified by PCR using primers (nptII_F/nptII_R) and an *FB_MR5* specific probe using primers (FbMr5_SondeF / FbMr5_SondeR, Table 1). Hybridization with each of the probes was performed using the ECF-Random-Prime-Labeling and Detection Kit (Amersham Biosciences, Freiburg, Germany) according to the manufacturer’s manual.

### Integration site of C44.4.146

Molecular characterization and iPCR for determination of insertion site was performed as described by Vanblaere et al. [45] with the following modifications: i) Backbone integration was assessed by PCR amplification (Fig 3) using primers Bb_LB1 and Bb_LB2 (Fig 1 and Table 1); ii) For iPCR, *Xba*I-digested DNA of cisgenic genotypes was subjected to ligase reaction and the primers iPCR-RB-XbaI-closer and iPCR-RBout (Table 1) were used to amplify by PCR the junction between T-DNA and genomic sequence at the right border (Fig 2). The resulting PCR product was then sequenced and BLAST analysis against the apple genome [59] was performed.

Primers IS146-1163F and IS146-1391R (amplicon E, Fig 5) that flank the insertion site were designed using the sequence of contig MDC012271.181 and used in combination with the primers IS146-LB-321R (amplicon F, Fig 5) or iPCR-RBout (amplicon G, Fig 5) to characterize the T-DNA junctions in the cisgenic line C44.4.146 (Fig 2). Primer sequences are summarized in Table 1.

### Transcription of *FB_MR5*

Transcript levels of *FB_MR5* in different genotypes were determined by RT-qPCR. A Taqman Probe with the sequence YYE-TGGCTTCCATTTCAAACGGATCACAGA-BHQ1 was designed to specifically detect *FB_MR5* in combination with primers FB_MR5q1 F and FB_MR5q1 R [56]. As reference primers pairs EF1α and relative Taqman probe developed by Gusberti et al. [60] were used. RNA was extracted from three young unfolded leaves of different plants of genotypes C44.4.146, 'Gala Galaxy' *in vitro*, and the classically bred *FB_MR5*-carrying accessions ACW 22161 and ACW 22176, using Zymo QuickRNA extraction kit (Zymo Research Corporation, Irvine, USA). Extracted RNA was then subjected to a second DNAse treatment (DNAse Ambion^®^ Life Technologies, Carlsbad, USA), after which first strand synthesis was performed using the Fermenta’s first strand minus H cDNA synthesis kit. cDNA was then diluted 1 / 10 and 5 μl were used for qPCR on ViiA Ruo qPCR device (Thermo Fisher scientific Inc. ©, Waltham, USA) in a total reaction volume of 20 μl using the Taqman Fast Universal Master Mix (Thermo Fisher Scientific Inc. ©, Waltham, USA). Each reaction had following primer / probe final concentration: EF1α primers at 600 nM, EF1α probe at 400 nM, FB_MR5q1 primers at 900 nM and FB_MR5q1 probe at 250 nM. The same reaction protocol was used to generate standard curves using both dilution series of DNA as well as cDNA from the cisgenic line C44.4.146. Relative expression ratio of *FB_MR5* / EF1α was calculated according to Pfaffl [61]. For transcription comparison we excluded three data points with CT_*FB_MR5*_ > 40 (two from ACW 22161 II) and one from ACW 22161 III)) as relative expression ratio in standard curves was no longer linear and standard deviation increased above a CT_*FB_MR5*_ value of 40.

### Statistical analysis

Statistical analysis was performed using software JMP^®^ 10.0 and 11.0 (SAS Institute INC., Cary, NC) and R [62]. As datasets did not follow normal distribution nonparametric tests were used to compare groups. Transcription level data were compared using the Steel Dwass test. In fire blight inoculation experiments 1 and 2, the Wilcoxon test was performed and in experiment 3 all groups were compared using the Steel Dwass test. Additionally all groups in all three experiments were compared simultaneously using the Steel Dwass test with corrected p-values (Fig 7). One outlier in experiment 2 (C44.4.146 inoculated by syringe) and one in experiment 3 ('Gala Galaxy' inoculated by scissors) were removed from the dataset for the statistical analysis as they differed more than 1.5 interquartile ranges from the corresponding group median (Fig 7). All statistical analysis were performed with a significance level of α = 0.05.

## Supporting Information

S1 Fig PCR tests to verify absence of backbone. An amplicon after PCR with primer pair Vf2_backbone_LB_1 and Vf2_backbone_LB_2 (Table 1) was observed in the transgenic lines T44.3 and T44.10 indicating presence of integrated backbone sequences beyond the left border (amplicon A, Fig 1). T45.5 did not amplify *FB_MR5* (data not shown) and was not further considered.
(TIF)

S2 Fig PCR tests to identify cisgenic lines. Amplicons after PCR with primer pair 167nptII-for / 367nptII-rev (Table 1) indicated that most of the investigated lines still carry *NptII* (amplicon B, Fig 1) and are therefore transgenic. Three cisgenic lines (239, 240 and 243) were identified and all three originated from the same transgenic motherline (T44.4) as C.44.4.146 (Fig 3).
(TIF)

S1 Table Overview of RT-qPCR results. Date when experiment was performed (date). Sample: ‘Gala Galaxy’ (Gala) Cisgenic line C44.4.146 (Cis), conventionally bred genotypes ACW 22161 (22161) and ACW 22176 (22176). Biological replicates are indicated (A, B or C). Target: Specific probe for Elongation Factor 1 α (EF1) or *FB_MR5* (Mr5). CT value: If no target was identified “Undetermined” is indicated. Transcription ratios were calculated in comparison to the average CT value of CisA on 4^th^ of May (21.209 for EF1 and 31.594 for Mr5) according to Pfaffl [61]. Gala plants showed no amplification with Mr5 (indicated in bold). *: Data was not included for Fig 6 according to experimental procedures.
(XLS)
