# Supplementary figures and images for: Development of the First Cisgenic Apple with Increased Resistance to Fire Blight
Source: PLoS One. 2015 Dec 1;10(12):e0143980. doi: 10.1371/journal.pone.0143980 (PMC4666654; doi:10.1371/journal.pone.0143980)

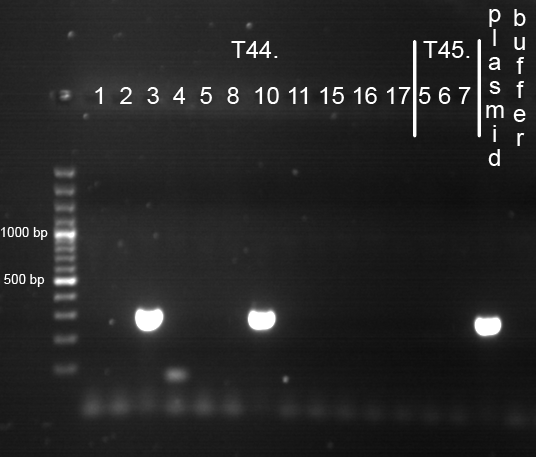

Supplement: S1 Fig — An amplicon after PCR with primer pair Vf2_backbone_LB_1 and Vf2_backbone_LB_2 (Table 1) was observed in the transgenic lines T44.3 and T44.10 indicating presence of integrated backbone sequences beyond the left border (amplicon A, Fig 1). T45.5 did not amplify FB_MR5 (data not shown) and was not further considered. (TIF) [file pone.0143980.s001.tif]

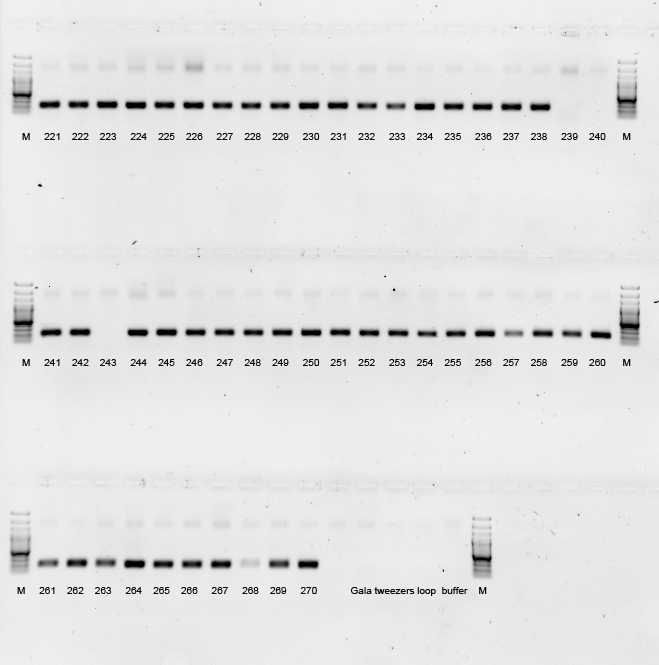

Supplement: S2 Fig — Amplicons after PCR with primer pair 167nptII-for / 367nptII-rev (Table 1) indicated that most of the investigated lines still carry NptII (amplicon B, Fig 1) and are therefore transgenic. Three cisgenic lines (239, 240 and 243) were identified and all three originated from the same transgenic motherline (T44.4) as C.44.4.146 (Fig 3). (TIF) [file pone.0143980.s002.tif]
